# High Prevalence of GES-5 Variant and Co-Expression of VIM-2 and GES-45 among Clinical *Pseudomonas aeruginosa* Strains in Tunisia

**DOI:** 10.3390/antibiotics12091394

**Published:** 2023-08-31

**Authors:** Meha Fethi, Beatriz Rojo-Bezares, Ameni Arfaoui, Raoudha Dziri, Gabriela Chichón, Farouk Barguellil, María López, Mohamed Selim El Asli, Paula Toledano, Hadda-Imen Ouzari, Yolanda Sáenz, Naouel Klibi

**Affiliations:** 1 Laboratory of Microorganisms and Active Biomolecules, Faculty of Sciences of Tunis, University of Tunis El Manar, Tunis 2092, Tunisia; 2 Área de Microbiología Molecular, Centro de Investigación Biomédica de La Rioja (CIBIR), 26006 Logroño, Spain; 3 Laboratory of Bacteriology, Military Hospital of Tunis, Tunis 1008, Tunisia; 4 Laboratory of Microorganisms and Environment, Molecular Diagnostic Tools and Emerging and Re-Emerging Infections (LR19DN03), Military Hospital of Tunis, Tunis 1008, Tunisia

**Keywords:** carbapenem resistance, GES-5, class 1 integron, high-risk clone ST235

## Abstract

Carbapenem-resistant *Pseudomonas aeruginosa* (CRPA) are a global health concern. The antimicrobial resistance, virulence, and molecular typing of 57 CRPA isolated from 43 patients who attended a specific Tunisian hospital from September 2018 to July 2019 were analyzed. All but one were multidrug-resistant CRPA, and 77% were difficult-to-treat-resistant (DTR) isolates. The *bla*_VIM-2_ gene was detected in four strains (6.9%), and among the 36 *bla*_GES_-positive CRPA (62%), the *bla*_GES-5_ gene was the predominant variant (86%). Three strains co-harbored the *bla*_VIM-2_ and *bla*_GES-45_ genes, and seven CRPA carried the *bla*_SHV-2a_ gene (14%). OprD alterations, including truncations by insertion sequences, were observed in 18 strains. Regarding the 46 class 1 integron-positive CRPA (81%), the *bla*_GES-5_ gene was located in integron In717, while the *bla*_GES-29_ and *bla*_GES-45_ genes were found in two new integrons (In2122 and In4879), and the *bla*_VIM-2_ gene was found in In1183 and the new integron In2142. Twenty-four PFGE patterns and thirteen sequence types (three new ones) were identified. The predominant serotype O:11 and *exoU* (81%) were mostly associated with ST235 and the new ST3385 clones. The seven *bla*_SHV-2a-_CRPA from different patients belonged to ST3385 and the same PFGE pattern. The *bla*_GES-5_- and *bla*_VIM-2_ + *bla*_GES-45_-positive CRPA recovered mostly from ICU patients belonged to the high-risk clone ST235. Our results highlight the alarming prevalence of *bla*_GES-5-_ and ST235-CRPA, the co-existence of *bla*_GES-45_ and *bla*_VIM-2_, and their location within integrons favoring their dissemination.

## 1. Introduction

*Pseudomonas aeruginosa* is a common Gram-negative pathogen that is recognized as a cause of several hospital-acquired infections ranging from urinary tract infections to life-threatening pneumonia and septicemia, especially among patients with cystic fibrosis, immunocompromised patients, burn patients, and those with indwelling devices. Carbapenems are considered as one of the last resort treatments of infections caused by *P. aeruginosa* [1,2]. Compared to other β-lactams, these potent compounds provide better Gram-negative coverage and exhibit stability against the action of extended spectrum β-lactamases (ESBL) and chromosomal cephalosporinase (AmpC), along with safety and efficiency [3,4]. However, this efficiency is increasingly impeded by the emergence of carbapenem-resistant *P. aeruginosa* (CRPA) isolates. Subsequently, the lack of therapeutic alternatives, together with the spread of CRPA involve that infections caused by this pathogen, have become difficult to treat and a cause of great concern, with significant consequences for clinical and economic outcomes [5,6]. Indeed, in 2017, the World Health Organization ranked CRPA in the critical group on the priority pathogens list, that imperatively requires the development of new antibiotics [7].

The mechanisms of resistance to carbapenem in *P. aeruginosa* include decreased drug uptake, loss of the OprD outer membrane porin, hyperexpression of efflux pump systems, overexpression of AmpC, and acquisition of carbapenemases. Furthermore, the combination of two or more of these carbapenem resistance mechanisms determines the level of carbapenem resistance [8,9,10,11]. Among the carbapenemases acquired by *P. aeruginosa,* the most important ones are metallo-beta-lactamases (MBL), mainly VIM, IMP, and NDM, but class A carbapenemases, including GES-type enzymes, less frequently KPC, and rarely class D oxacillinases (OXA-type), are also responsible for carbapenem resistance [5,12,13,14]. The 59 known GES variants (http://www.bldb.eu, accessed on 28 August 2023) [15] are categorized as minor ESBLs, with the exception of the variants that possess amino acid substitutions within their active sites (Gly170Ser or Gly170Asn) (such as GES-5) because they can expand their spectrum of activity against carbapenems and are categorized as carbapenemases [16]. GES-5-producing *P. aeruginosa* was first described in China [17] and has been increasingly detected worldwide (e.g., South Africa, Brazil, Mexico, Spain, Turkey, Saudi Arabia, and Japan), with it even being associated with the *P. aeruginosa* ST235 high-risk clone [14,18]. Carbapenemase-encoding genes are generally located on mobilized genetic elements such as plasmids, transposons, and integrons; they include numerous antimicrobial resistance determinants, thus promoting the dissemination of multidrug-resistance [14,19].

In addition to its great antimicrobial resistance, the importance of *P. aeruginosa* is marked by the presence of multiple virulence factors [20], most of them under the control of the quorum-sensing system (mainly LasI/LasR and RhlI/RhlR systems), which is a cell density recognition mechanism. The Type 3 Secretion System (T3SS) and its toxins, termed effectors (ExoU, ExoS, ExoT, ExoY), are the major virulence determinants in *P. aeruginosa*. These systems enable *P. aeruginosa* to invade and infect the host, thereby increasing pathogenicity [21]. ST235 is the most globally disseminated of the three major international high-risk clones (ST111, ST175, and ST235), and it exhibits a highly virulent phenotype with a high mortality rate, which is most likely due to the production of the ExoU effector [22].

Based on the alarming aspect of multidrug-resistant *P. aeruginosa* strains, molecular typing is essential to investigate the diversity of *P. aeruginosa* collections and to confirm or deny the genetic relationship between the strains during outbreaks. In fact, methods such as Pulsed field gel electrophoresis (PFGE) and Multilocus sequence typing (MLST) are valuable tools for identifying the sources of infection and putative reservoirs and tracking the transmission routes of high-risk clones within and across wards, hospitals, cities, and even countries [23,24].

In Tunisia, rates of carbapenem-resistant Gram-negative bacteria are steadily increasing. This country was the second largest consumer of antibiotics in the world in 2015, and according to the Pharmacy and Medicines Directorate, carbapenem consumption doubled between 2011 and 2015 because of the overuse and misuse of antibiotics in human medicine and the livestock, agricultural and industrial sectors. Thus, the CRPA rates reached 46% of *P. aeruginosa* strains isolated in the ICU, and nearly 42% of these strains were extensively drug-resistant (http://www.dpm.tn/, accessed on 1 July 2023). Regarding CRPA studies in Tunisia, outbreaks of VIM-producing *P. aeruginosa* strains and, more recently, GES-5-producing CRPA have been reported [25,26,27,28,29,30,31]. Additionally, the successful worldwide spread of high-risk clones of *P. aeruginosa* poses a threat to global public health that needs to be studied and managed. However, few reports have been conducted on the molecular epidemiology or molecular typing of CRPA in Tunisia [29,30,31]. The aim of the present study was to analyze the characteristics of clinical CRPA isolates recovered from the Military Hospital of Tunis, including their antimicrobial resistance, integrons, virulence factors, and molecular typing.

## 2. Results

### 2.1. Epidemiological Results

During the sampling period, a total of 290 *P. aeruginosa* isolates were recovered from clinical samples in the Military Hospital of Tunis, and 57 of them were CRPA (20%). These strains were obtained from various clinical samples (as shown in Table 1), such as the following: tracheal aspirate (*n* = 23), bronchoalveolar lavage (*n* = 5), sputum (*n* = 2), catheter (*n* = 4), blood (*n* = 13), pus (*n* = 7), urine (*n* = 2), and urethral samples (*n* = 1) (Supplementary Table S1). The highest percentage was respiratory isolates (53%), followed by isolates from blood cultures (23%) and pus (12%). Most of the CRPA strains (82.4%) were recovered from intensive care unit (ICU) patients, although isolates from patients of other departments, including vascular surgery, orthopedic surgery, cardiothoracic surgery, urology, and bacteriology, were obtained. These CRPA were mainly isolated from male patients (79%) aged between 27 and 86 years (average age: 50 years) (Table 1).

### 2.2. Antimicrobial Susceptibility and Phenotypic Tests

Antimicrobial susceptibility testing showed that all strains had a high minimum inhibitory concentration (MIC) of imipenem (≥16 µg/mL), and most of them were also resistant to meropenem (97%) (Table 2). Moreover, all CRPA but P36 were multidrug-resistant (MDR), 44 strains were difficult-to-treat resistant (DTR), and 40 strains were resistant to all antipseudomonal agents tested (70%). The following high percentages of antimicrobial resistance were detected: aztreonam (100%), ticarcillin-clavulanic acid (98%), piperacillin (90%), piperacillin-tazobactam (77%), cefepime and ceftazidime (83%), aminoglycosides (75–83%), ciprofloxacin and levofloxacin (86%). The ESBL phenotype was observed in seven isolates (12%), in which, after confirmation via PCR and sequencing, the *bla*_SHV-2a_ gene was detected. The MBL phenotype was identified in four strains (7% of CRPA) linked to the presence of the *bla*_VIM-2_ gene, and additionally, three of them co-harbored the *bla*_VIM-2_ and *bla*_GES-45_ genes. The inducible AmpC test was positive for 38 isolates, AmpC hyperproduction was detected in 5 strains (7%), and efflux pump overexpression was observed in 51 CRPA (89%) (Supplementary Table S1). Efflux pump overexpression in the presence of ciprofloxacin, imipenem, and meropenem was noted with percentages of 81%, 39%, and 26% of CRPA isolates, respectively.

### 2.3. Characterization of Carbapenem Resistance

The molecular characterization of CRPA showed that 35 of them were carbapenemase producers with a predominance of GES-5 (31/35) against a low prevalence of VIM-2 (4/35). Likewise, the following non-carbapenemase GES-type enzymes were detected in five isolates: GES-1, GES-29, and GES-45 (Table 2). As Table 1 shows, the patients with carbapenemase-CRPA carriers were all admitted to the ICU, except for one who required orthopedic surgery, whereas the remaining patients were distributed in different hospital wards.

The *oprD* gene was amplified from all but two of the fifty-seven CRPA (P22 and P70), and sixteen amplicons were randomly selected for sequencing. Table 3 shows the high polymorphism in OprD amino acid sequences. Amino acid substitutions and nucleotide insertions and deletions were detected, highlighting the presence of the insertion sequences IS*Pa33* and IS*Pa26*, which disrupt the *oprD* gene of P4 and P569, respectively (Table 3).

### 2.4. Molecular Typing and Serotypes of CRPA Isolates

**Figure 1:**
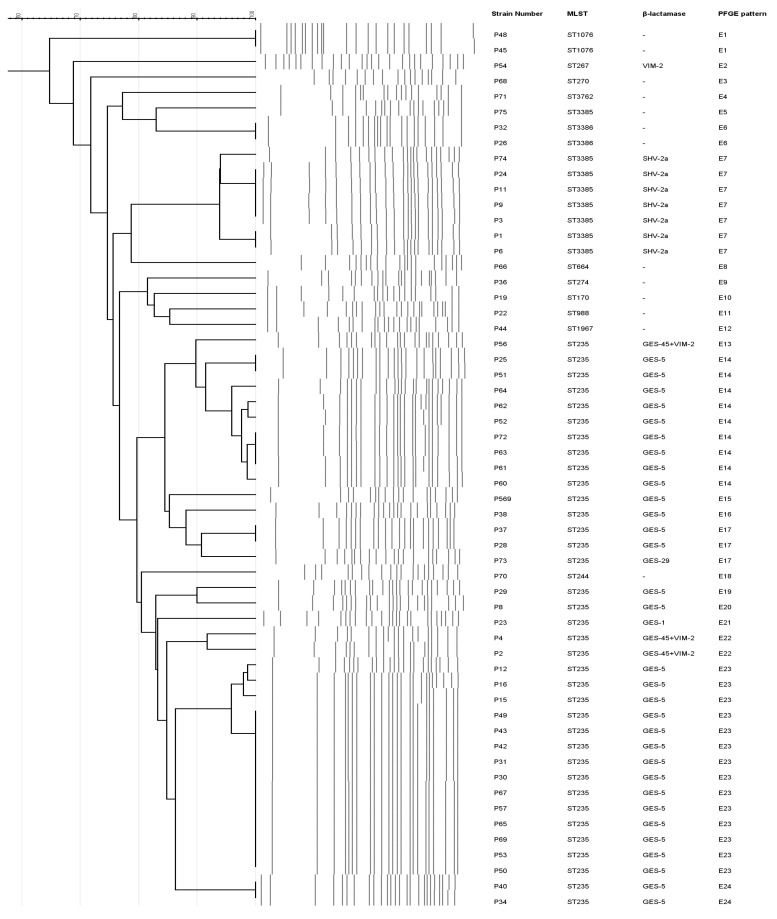
PFGE, MLST, and β-lactamase genes of the 57 CRPA isolates.

PFGE revealed 24 distinct PFGE patterns among the 57 isolates, and MLST showed the presence of 13 different sequence types (Table 2 and Figure 1). Thirty-six isolates (63% CRPA) belonged to the high-risk clone ST235. Three novel sequence types—ST3385, ST3386, and ST3762—were first described in this study and named by the PubMLST database. The remaining isolates were assigned to ST170, ST244, ST267, ST270, ST274, ST664, ST988, ST1076, and ST1967 (Figure 1). The most frequent serotype was O:11 (82%); although four CRPA were non serotypable (two polyagglutinable and two non-agglutinable), the remaining CRPA were serotyped as O:16, O:5, O:4, or O:2 (Table 2).

It is worrying to highlight that the seven *bla*_SHV-2a_-harboring CRPA were isolated from seven different patients of three wards, but all of them belonged to ST3385 and to the same E7 PFGE pattern. Moreover, the 31 *bla*_GES-5_-positive CRPA which were recovered from 21 patients admitted to the ICU (except one from a patient who underwent an orthopedic surgery) were serotyped as O:11, grouped in 8 different PFGE patterns (E14, E15, E16, E17, E19, E20, E23, E24), and belonged to the high-risk clone ST235 (Supplementary Table S1). However, the four *bla*_VIM-2_ carriers recovered from four different patients were grouped in two MLST (O:11-ST235 and O:16-ST267) and three PFGE (E2, E13, E22) (Supplementary Table S1).

### 2.5. Detection of Virulence Factors

The presence of the seven genes involved in the virulence and quorum-sensing system was investigated among the 57 *P. aeruginosa* strains, and six different profiles were obtained (Table 4). The T3SS *exoU^+^/exoS*^-^ genotype was detected in 46 isolates (81%), and the *exoU^-^/exoS*^+^ genotype was detected in the remaining 11 isolates (19%). On the other hand, no isolate produced the exolysin-encoding gene *exlA*. All strains amplified the *lasI* gene, but the *lasR* gene was amplified in 52 CRPA (91%). P6 and P74 CRPA showed a <400 bp *lasR* amplicon. On the other hand, the *lasR* amplicon of the P38 strain was larger than 2 kb, and sequencing analysis showed that the insertion sequence IS*Pa26* truncated the *lasR* gene. The *rhlI* gene was absent in six strains, and the *rhlR* was absent in 11 CRPA.

### 2.6. Detection and Characterization of Integron Structures

Class 1 integrons were detected in 46 of the 57 CRPA isolates (81%), but class 2 and class 3 integrons were not found (Supplementary Table S1). Four new class 1 integrons were detected in this study, namely In2115, In2122, In2142, and In4879 (named by INTEGRALL), and were submitted to GenBank with the following respective accession numbers: OM831263, OM831264, OM863779, and OQ858935.

**Figure 2:**
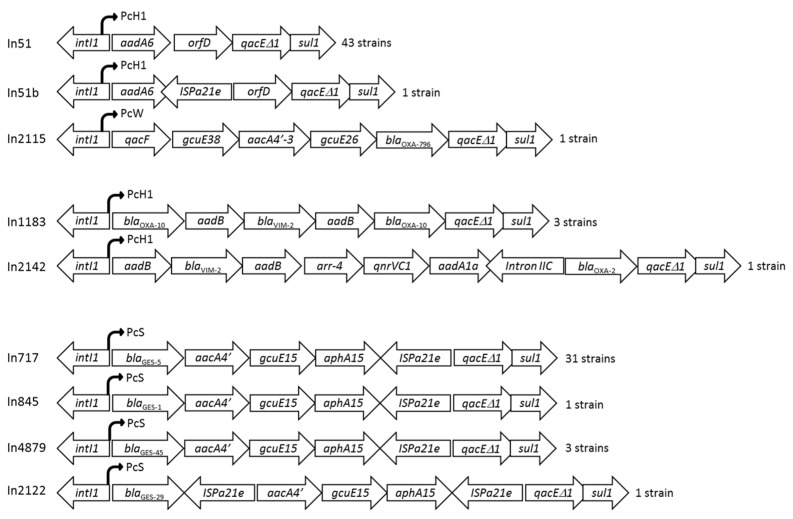
Schematic structure of the different class 1 integrons detected in the 57 CRPA.

More than one integron per isolate was detected in 78% of the isolates (36/46), and all of them were ascribed to the high-risk clone ST235. The carbapenemase-producing CRPA harbored three types of class 1 integrons containing either *bla*_VIM-2_ (In1183 and In2142 regulated by a hybrid 1 promoter (PcH1)) or *bla*_GES-5_ (In717 regulated by a strong promoter (PcS)) (Figure 2). The In2142 presented a new genetic arrangement (*aadB* + *bla*_VIM-2_ + *aadB* + *arr*-4 + *qnrVC1* + *aadA1a* + intronIIC + *bla*_OXA-2_) and was regulated by a PcH1 promoter. The *bla*_GES-1_, *bla*_GES-29_, and *bla*_GES-45_ genes were located as gene cassettes within the class 1 integrons (In845, In2122, and In4879, respectively) and were composed by the same genetic arrangements that included genes involved in aminoglycoside resistance (*aacA4′*, *aphA15*). The remaining isolates contained genetic arrangements not associated with carbapenem resistance, such as the integron In2115 (*qacF* + *gcuE38* + *aacA4′-3* + *gcuE26* + *bla*_OXA-796_) and integrons In51 and In51b (Figure 2).

## 3. Discussion

The emergence and propagation of MDR and DTR *P. aeruginosa* strains is a global burden that makes hospital-acquired infections challenging to treat due to the lack of effective and safe therapeutic options. This worrisome situation has prompted more investigations to better understand and monitor multidrug resistance trends [7,8]. Carbapenems are used to treat infections, but the rates of carbapenem resistance and DTR *P. aeruginosa* vary worldwide [2,14]. Our study revealed that 20% (57/290) of the *P. aeruginosa* isolates collected from the patients of a Tunisian hospital were carbapenem-resistant, with 98% (56/57) of them being MDR, 77% being DTR, and 70% being resistant to all antipseudomonal agents tested. Similar rates of CRPA have been reported in Spain (16–19%), Algeria (19%), the United States and South Africa (21%), and Dubai (24%) [14,32,33,34]. This incidence, however, is lower than the figures previously reported in Tunisia (33–54%) [29,35] and in other countries, such as those in South America (31%), Egypt (59%), Saudi Arabia (52%), and Lithuania (>60%) [14,36,37,38].

In the current study, most of the CRPA were found in respiratory specimens from ICU patients. Typically, MDR bacteria are mostly recovered in the ICU, where the extensive use of antibiotics, particularly carbapenems, creates selection pressure, that promotes the emergence of MDR strains [39,40]. In addition, our data highlighted the high percentage of DTR-*P. aeruginosa* (77%), whose treatment requires new options such as ceftolozane-tazobactam, ceftazidime-avibactam, or imipenem-relebactam, as international guidelines currently recommend [41].

Our analysis of the carbapenem resistance mechanisms in the 57 CRPA revealed that carbapenemases (GES-5 and VIM-2), active efflux pumps, the inducible expression of AmpC, and the overexpression of AmpC β-lactamase were found in 61%, 89%, 67%, and 7% of the *P. aeruginosa* isolates, respectively. Additionally, a high polymorphism in the porin OprD was observed in the 18 CRPA analyzed. As previously reported [42,43,44,45,46], the detected insertions, deletions, premature stop codons, and insertion sequences IS*Pa33* and IS*Pa26* truncating the *oprD* gene would suppose a direct correlation with carbapenem resistance, mainly imipenem resistance, by disrupting the coding region of the *oprD* gene or by downregulating its expression in our CRPA. The combination of two or more of these carbapenem resistance mechanisms determined the carbapenem resistance in the studied CRPA.

Reports of various carbapenemase types among *P. aeruginosa* strains have increased worldwide over the last decade [14,15,47]. GES-5 class A carbapenemase (54%) appears to be the leading carbapenemase in this study. Moreover, four isolates (7%) were VIM-2 MBL producers. This result aligns with the recent studies reporting the detection of GES-5 and VIM-2 in the CRPA of Tunisia [30,31], and it is worth mentioning that this GES-5 prevalence in CRPA might be a sign of the start of the expansion of these strains in Tunisia. On the other hand, the predominance of VIM-2 among *P. aeruginosa* was previously revealed in studies performed in Tunisia [25,28,29,31] and in other countries such as the United Arab Emirates [34], Spain [32,43,44,48], Russia, the United States [14], Lebanon, and Egypt [49,50].

Along with the detected carbapenemases, GES-1, GES-29, and GES-45 class A β-lactamases and the SHV-2a ESBLs were also found in our study. Indeed, three *bla*_VIM-2_-producing isolates also harbored the *bla*_GES-45_ gene. The co-existence of VIM and GES enzymes in *P. aeruginosa* has been previously demonstrated in Tunisia [30,31] as well as elsewhere [34,51,52]. However, to our knowledge, this is the first work to describe the co-existence of the *bla*_GES-45_ and *bla*_VIM-2_ genes in CRPA.

GES-5-producing *P. aeruginosa* clones have been identified worldwide [14,48,53]. In our work, MLST and PFGE enabled us to compare and establish the genetic link between CRPA isolates and revealed that the 31 GES-5-producing CRPA clustered together and belonged to the internationally spread high-risk clone ST235 and 8 different PFGE patterns. In accordance with previous studies [18,22,32,38,48,54,55], our results showed that ST235 was associated with MDR, the *exoU* gene, and GES-5-producing and VIM-2-, GES-1-, GES-29-, and GES-45-producing isolates. Indeed, 63% of the CRPA were ascribed to ST235, the high-risk clone reported in hospital outbreaks worldwide and associated with MDR patterns via the acquisition of carbapenemases [18,48,54].

*P. aeruginosa* can also exhibit resistance to cephalosporins mediated by ESBLs embedded in plasmids and/or integrons. In fact, GES- and SHV-type ESBLs mainly possess ceftazidimase activity, affecting cefepime to a lesser extent [56]. In our work, particularly noteworthy was the existence of seven MDR *bla*_SHV-2a_-producing CRPA belonging to the new *exoU*-positive ST3385 and to the same E7 PFGE pattern, which spread in different wards of the hospital. These results suggest an intra-hospital outbreak due to a SHV-2a-producing CRPA, as previously described by other authors in France and Tunisia [25,57].

Along with the ST235 high-risk clone, ST244 and ST274 are of particular significance as they are frequently detected worldwide but not always linked to MDR/XDR profiles, as our results show [58].

Concerning *P. aeruginosa* pathogenicity, in our study, 81% of the CRPA were *exoU*-positive strains with O:11 or polyagglutinable serotypes and belonged to ST235 (78%), ST3385 (18%), and ST1076 (4%). Infections related to *exoU*-producing strains have been found to be associated with more severe clinical symptoms and poorer outcomes than infections caused by *exoS^+^/exoU^-^* isolates [44,59,60,61]. Moreover, MDR *P. aeruginosa* strains, especially CRPA, also contribute to poorer clinical outcomes.

Several studies have revealed that high levels of antimicrobial resistance are represented by a relatively low genetic diversity, as demonstrated by the current work [14,32,58]. However, the horizontal gene transfer of carbapenemase genes through mobilizable genetic elements such as integrons requires special attention because it can accelerate the dissemination of MDR CRPA [14,62]. Class 1 integrons were detected in 81% of our CRPA isolates, with 9 different gene cassette arrangements containing genes encoding resistance to β-lactams, aminoglycosides, rifampicin, and fluoroquinolones. More than one integron per isolate was detected in 78% of the CRPA, as described in other studies [43], and all of them were ascribed to ST235. Four new genetic arrangements were detected in the study (In2115, In2122, In2142, In4879), and all of the carbapenemase genes were detected as gene cassettes embedded in class 1 integron structures. Three of the four *bla*_VIM-2_-positive CRPA harbored the In1183 integron (*bla*_OXA-10_ + *aadB* + *bla*_VIM-2_ + *aadB* + *bla*_OXA-10_), an arrangement first described in Tunisia [29]. Interestingly, a new arrangement (*aadB* + *bla*_VIM-2_ + *aadB* + *arr-4* + *qnrVC1* + *aadA1a* + intronIIC + *bla*_OXA-2_) was identified in the *exoS*-VIM-2-positive isolate belonging to ST267. This integron partially shared the arrangement of a class 1 integron also first described in Tunisia (*aadB* + *bla*_VIM-2_ + *aadB* + *arr-6* + *qnrVC1* + *aadA1c* + intronIIC + *bla*_OXA-2_) [63]. Additionally, all 31 GES-5-positive isolates carried a previously described arrangement (*bla*_GES-5_ + *aacA4′* + *gcuE15* + *aphA15* + IS*Pa21e*) [18].

This is the first study to show the coexistence of VIM-2 and GES-45 and elucidate the phylogenetic relationship (by PFGE and MLST) as well as the main virulence determinants among circulating CRPA strains in Tunisia. However, a limitation of our study is that it was performed using CRPA from a single hospital. Thus, the prevalence and molecular characteristics of the CRPA strains may not be nationally representative. Moreover, the absence of susceptibility testing to last-line and novel antimicrobials (e.g., ceftolozane-tazobactam, ceftazidime-avibactam, and imipenem-relebactam), together with the several difficulties experienced in obtaining more clinical patient data, such as length of stay, comorbidities, outcome (recovery or death), etc., meant that we were unable to analyze the impact of the high rates of DTR *P. aeruginosa* and CRPA on the clinical outcomes of patients.

## 4. Materials and Methods

### 4.1. Bacterial Isolates and Identification

A total of fifty-seven non-duplicated CRPA isolates were recovered from forty-three patients of the Military Hospital of Tunis from September 2018 to July 2019. Bacterial identification was firstly performed via the Vitek^®^2 automated system (Biomérieux, Marcy-l’Étoile, France), then confirmed via PCR amplification of *oprL*, the outer cell membrane lipoprotein L-encoding gene [64], and by Matrix-Assisted Laser Desorption/Ionization Time-of-Flight Mass Spectrometry (MALDI-TOF MS) (MALDI Biotyper^®^, Bruker Daltonics GmbH & Co. KG, Bremen, Germany) following the manufacturer’s guidelines. The results were interpreted according to the MBT Compass Library DB-6903 (V.6).

### 4.2. Antimicrobial Susceptibility Tests

Susceptibility testing to 13 antipseudomonal agents, including ticarcillin-clavulanic acid (TCC), piperacillin, piperacillin-tazobactam, ceftazidime (CAZ), cefepime (FEP), aztreonam, imipenem (IMP), meropenem (MEM), amikacin, gentamicin, tobramycin, ciprofloxacin (CIP), and levofloxacin was carried out using the Vitek^®^2 compact analyser (Biomérieux) and interpreted according to the European Committee on Antimicrobial Susceptibility Testing (EUCAST) breakpoints (2020) [65]. For the purposes of this study, EUCAST v9.0 breakpoints were used for gentamicin. *P*. *aeruginosa* isolates that were non-susceptible to at least one agent in three or more antimicrobial categories were considered multidrug-resistant (MDR) [66], and those isolates non-susceptible to all of the following antibiotics were considered difficult-to-treat resistant (DTR): piperacillin–tazobactam, ceftazidime, cefepime, aztreonam, meropenem, imipenem, ciprofloxacin, and levofloxacin [2,41]. The MBL phenotype was screened via the double-disc synergy test (IMP, 0.5 M EDTA (pH 8), MEM) [67], as were the ESBL (FEP disc close to amoxicillin + clavulanic acid disc) and inducible AmpC (IMP disc close to CAZ) phenotypes. AmpC hyperproduction was analyzed using CAZ discs and Mueller-Hinton agar plates in the presence or absence of cloxacillin (250 mg/L). Likewise, efflux pump overexpression was studied using IMP, MEM, and CIP discs located on Mueller-Hinton agar plates with or without the Phe-Arg-β-naphthylamide inhibitor (PaβN, 40 mg/L). AmpC hyperproduction and efflux pump overexpression were evidenced by an increase in the inhibition zones of at least 5 mm [68].

### 4.3. Molecular Typing

The clonal relationship among the fifty-seven recovered CRPA isolates was determined via PFGE. Agarose plugs containing *SpeI*-restricted genomic DNA were prepared as previously described [69], and DNA fragments were separated using two ramps at 6 V/cm at 14 °C within a CHEF-DR II system (BioRad). The pulse time ranged from 5 to 15 s during the first 10 h and from 15 to 45 s during the next 10 h. A lambda ladder (Bio-Rad) was used as a DNA size marker. The DNA profiles were analyzed using the GelJ software 2.3 (UPGMA algorithm; Dice coefficient) [70]. The isolates showing a Dice coefficient ≥90% were considered genetically related for this study.

Multilocus Sequence Typing MLST was performed by amplification and subsequent bidirectional sequencing of seven housekeeping genes (*acsA, aroE, guaA, mutL, nuoD, ppsA,* and *trpE*). The nucleotide sequences of alleles were compared with those of the PubMLST database to assign allele numbers and STs (http://pubmlst.org/paeruginosa/, accessed on 21 October 2019) [24]. Newly detected STs were submitted to the PubMLST website.

### 4.4. Serotyping

Serotypes of the recovered isolates were determined via the slide agglutination test (Bio-Rad, Temse, Belgium), which was conducted according to the International Antigenic Typing scheme and by using 16 type O monovalent antisera specific for *P. aeruginosa* following the manufacturer’s protocol.

### 4.5. Characterization of Class A Carbapenemases, MBLs, ESBL, and Porin OprD

The molecular screening of β-lactamase genes was carried out via PCR sequencing. The presence of genes encoding class A β-lactamases (*bla*_GES_, *bla*_IMI_, *bla*_SME_, *bla*_KPC_), MBLs (*bla*_IMP_, *bla*_VIM_, *bla*_SPM_, *bla*_SIM_, *bla*_GIM_, *bla*_NDM_, *bla*_BIC_, *bla*_DIM_, and *bla*_AIM_), and ESBLs (*bla*_TEM_, *bla*_SHV_, *bla*_CTX-M_, *bla*_PER_, *bla*_VEB_, and *bla*_BEL_) were investigated via PCR using specific primers [71,72,73,74,75]. Sequencing was performed to determine the gene variants.

OprD porin analysis was carried out via PCR and subsequent sequencing [71]. The mutations were determined by comparison with the wild-type *P. aeruginosa* strain PAO1 sequence (GenBank accession number AE004091) [76,77], and the insertion sequences were characterized using the online tool ISfinder (https://www-is.biotoul.fr/, accessed on 7 November 2019).

### 4.6. Detection and Characterization of Integrons

The presence of genes encoding type 1, 2, and 3 integrases, 3′-conserved segment of class 1 integrons (*qacE*Δ1+ *sul1*), and Tn402 features was studied via PCR. The characterization of class 1 integron variable regions and gene cassette promoters (Pc) was carried out via PCR mapping and sequencing [78].

### 4.7. Detection of Virulence Factors

All CRPA isolates were tested for the presence of virulence markers such as *exoS* and *exoU* exotoxin, *exlA* exolysin genes, and the quorum-sensing *lasR, lasI, rhlR*, and *rhlI* genes [79,80]. All the performed PCRs in this work included at least one positive control.

## 5. Conclusions

In summary, our findings highlight that GES-5 production, the co-existence of *bla*_GES-45_ and *bla*_VIM-2_, and OprD porin alterations were the major causes of carbapenem resistance in the CRPA strains. In our study, the GES-5-producing strains, which were mainly recovered from ICU patients, belonged to clonal clusters and to the international high-risk clone ST235. Moreover, given the pivotal role of integrons in the acquisition and transmission of antibiotic resistance genes, the prevalence of these elements in our isolates is a cause of concern and speaks to the need to implement policies to control the spread of resistance determinants. Based on the above, our findings could be invaluable to future research on the risk factors, origins, and mechanisms underlying outbreaks, thereby also being invaluable to the control of DTR *P. aeruginosa*.

## Figures and Tables

**Table 1:** Clinical characteristics of the CRPA isolates recovered between 2018 and 2019.

Characteristic	Number of CRPA (*n* = 57)	Carbapenemase Producer (*n* = 35)	Non-Carbapenemase Producer (*n* = 22)
Patient Gender:			
Number of males (%)	45 (79%)	25 (56%)	20 (44%)
Number of females (%)	12 (21%)	10 (83%)	2 (17%)
Patient age (year range)			
[20–45]	16 (28%)	9 (56%)	7 (44%)
[46–64]	19 (33%)	12 (63%)	7 (37%)
[65–80]	22 (39%)	14 (64%)	8 (36%)
Hospital admission to:			
Intensive care unit	47 (82%)	34 (72%)	13 (28%)
Vascular surgery	4 (7%)	0 (0%)	4 (100%)
Cardiothoracic surgery	2 (3.5%)	0 (0%)	2 (100%)
Orthopedic surgery	2 (3.5%)	1 (50%)	1 (50%)
Urology	1 (2%)	0 (0%)	1 (100%)
Bacteriology	1 (2%)	0 (0%)	1 (100%)
Type of sample:			
Tracheal aspirate	23 (40%)	16 (70%)	7 (30%)
Broncho-alveolar lavage	5 (9%)	4 (80%)	1 (20%)
Sputum	2 (3.5%)	0 (0%)	2 (100%)
Blood	13 (23%)	9 (69%)	4 (31%)
Pus	7 (12%)	1 (14%)	6 (86%)
Catheter	4 (7%)	3 (75%)	1 (25%)
Urine	2 (3.5%)	1 (50%)	1 (50%)
Urethral sample	1 (2%)	1 (100%)	0 (0%)

**Table 2:** Resistance phenotypes, beta-lactam genotypes, and molecular typing of 57 CRPA clinical isolates.

Strains	Resistance Phenotype	MIC (mg/L)		Beta- Lactamases	Molecular Typing		
		IPM	MER		MLST	PFGE Pattern (nº. Strains)	Serotype
P8, P12, P15, P16, P25, P28, P29, P30, P31, P34, P37, P38, P40, P42, P43, P49, P50, P52, P53, P57, P60, P61, P62, P63, P64, P67, P69, P72, P569	IPM, MEM, CAZ, FEP, TCC, PIP, PTZ, ATM, AMK, GEN, TOB, CIP, LVX	≥16	≥16	GES-5	ST235	E14 (8), E15 (1), E16 (1), E17 (2), E19 (1), E20 (1), E23 (13), E24 (2)	O:11
P51	IPM, MEM, CAZ, FEP, TCC, PIP, ATM, AMK, GEN, TOB, CIP, LVX	≥16	≥16	GES-5	ST235	E14 (1)	O:11
P65	IPM, MEM, CAZ, TCC, PIP, ATM, AMK, GEN, TOB, CIP, LVX	≥16	≥16	GES-5	ST235	E23 (1)	O:11
P23	IPM, MEM, CAZ, FEP, TCC, PIP, PTZ, ATM, AMK, GEN, TOB, CIP, LVX	≥16	8	GES-1	ST235	E21 (1)	O:11
P73	IPM, MEM, CAZ, FEP, TCC, PIP, ATM, GEN, TOB, CIP, LVX	≥16	4	GES-29	ST235	E17 (1)	O:11
P2, P4, P56	IPM, MEM, CAZ, FEP, TCC, PIP, PTZ, ATM, AMK, GEN, TOB, CIP, LVX	≥16	≥16	VIM-2, GES-45	ST235	E13 (1), E22 (2)	O:11
P54	IPM, MEM, CAZ, FEP, TCC, PIP, PTZ, ATM, GEN, TOB, CIP, LVX	≥16	≥16	VIM-2	ST267	E2 (1)	O:16
P1, P3, P9, P11, P24, P74	IPM, MEM, CAZ, FEP, TCC, PIP, PTZ, ATM, AMK, GEN, TOB, CIP, LVX	≥16	4–8	SHV-2a	ST3385	E7 (6)	O:11
P6	IPM, MEM, CAZ, FEP, TCC, PIP, PTZ, ATM, GEN, TOB, CIP, LVX	≥16	8	SHV-2a	ST3385	E7 (1)	PA
P19	IPM, MEM, TCC, ATM	≥16	4	-	ST170	E10 (1)	Agg-
P22	IPM, MEM, TCC, ATM	≥16	4	-	ST988	E11 (1)	O:4
P26	IPM, MEM, CAZ, FEP, TCC, PIP, PTZ, ATM, CIP, LVX	≥16	≥16	-	ST3386	E6 (1)	O:5
P32	IPM, MEM, CAZ, FEP, TCC, PIP, PTZ, ATM, GEN, CIP, LVX	≥16	≥16	-	ST3386	E6 (1)	PA
P36	IPM, MEM, ATM	≥16	4	-	ST274	E9 (1)	Agg-
P44	IPM, MEM, TCC, ATM	≥16	4	-	ST1967	E12 (1)	O:11
P45, P48	IPM, MEM, TCC, PIP, ATM	≥16	≥16	-	ST1076	E1 (2)	O:11
P66	IPM, MEM, FEP, TCC, PIP, ATM, AMK, GEN, TOB, CIP, LVX	≥16	≥16	-	ST664	E8 (1)	O:5
P68	IPM, MEM, TCC, PIP, ATM, CIP, LVX	≥16	≥16	-	ST270	E3 (1)	O:16
P70	IPM, TCC, ATM	≥16	2	-	ST244	E18 (1)	O:2
P71	IPM, TCC, ATM	≥16	2	-	ST3762	E4 (1)	O:11
P75	IPM, MEM, CAZ, FEP, TCC, PIP, PTZ, ATM, AMK, GEN, TOB, CIP, LVX	≥16	≥16	-	ST3385	E5 (1)	O:11

IPM, imipenem; MEM, meropenem; CAZ, ceftazidime; FEP, cefepime; TCC, ticarcillin-clavulanic acid; PIP, piperacillin; PTZ, piperacillin-tazobactam; ATM, aztreonam; AMK, amikacin; GEN, gentamicin; TOB, tobramycin; CIP, ciprofloxacin; LVX, levofloxacin. -: no beta-lactamase was detected. Agg-: no agglutinable, PA: polyagglutinable.

**Table 3:** Molecular characterization of porin OprD in 18 CRPA isolates.

Strains	MLST	OprD Size (Amino Acid)	Amino Acid Changes in OprD Sequence	Insertion/Deletion	MIC (mg/L)		Beta- Lactamase
					IPM	MEM	
P4	ST235	-	-	OprD is truncated by IS*Pa33*	≥16	≥16	VIM-2, GES-45
P45	ST1076	>443	T103S, K115T, F170L, E185Q, P186G, V189T, R310E, A315G	insertion of 1 bp (C) at nt 1205	≥16	≥16	-
P51	ST235	340	T103S, K115T, F170L, E185Q, P186G, V189T	deletion of 13 bp at nt 836	≥16	≥16	GES-5
P56	ST235	283	T103S, K115T, F170L, E185Q, P186G, V189T, Y282V, T283STOP	deletion of 59 bp at nt 845	≥16	≥16	VIM-2, GES-45
P66	ST664	93	-	deletion of 1 bp (C) at nt 198	≥16	≥16	-
P569	ST235	-	-	OprD is truncated by IS*Pa26*, and lacks ATG (M1)	≥16	≥16	GES-5
P1, P11, P74, P75	ST3385	237	T103S, K115T, F170L	deletion of 1 bp (G) at nt 557	≥16	8	SHV-2a
P19	ST170	345	D43N, S57E, S59R, E202Q, I210A, E230K, S240T, N262T, A267S	deletion of 1 bp (C) at nt 825	≥16	8	-
P26	ST3386	164	-	deletion of 11 bp at nt 209	≥16	≥16	-
P36	ST274	348	D43N, S57E, S59R, E202Q, I210A, E230K, S240T, N262T, A267S, A281G, K296Q, Q301E, R310G, S349STOP	-	≥16	2	-
P44	ST1967	433	T103S, K115T, F170L	-	≥16	2	-
P68	ST270	162	-	deletion of 17 bp at nt 358	≥16	≥16	-
P71	ST3762	467	D43N, S57E, S59R, E202Q, I210A, E230K, S240T, N262T, A267S, A281G, K269Q, Q301E, R310G, V359L	Loop7-short ^a^, insertion of 2 bp (TC) at nt 1322	≥16	2	-
P22 ^b^	ST988	-	-	-	≥16	2	-
P70 ^b^	ST244	-	-	-	≥16	2	-

^a^ Shortening of putative Loop L7: 372V-DSSSSYAGL-L383. ^b^ No amplicon of the *oprD* gene was detected in these strains.

**Table 4:** Virulence and quorum-sensing genes detected among the 57 CRPA isolates.

Strains	Virulence Factors						
	*exoS*	*exoU*	*exlA*	*lasI*	*lasR*	*rhlI*	*rhlR*
P19, P22, P26, P32, P36, P44, P54, P66, P71	+	−	−	+	+	+	+
P68, P70	+	−	−	+	+	−	+
P8, P12, P15, P16, P23, P25, P28, P29, P30, P31, P34, P37, P40, P42, P43, P45, P48, P49, P50, P51, P52, P53, P57, P60, P61, P62, P63, P64, P65, P67, P69, P72, P73, P569	−	+	−	+	+	+	+
P38	−	+	−	+	− ^a^	+	+
P1, P3, P9, P11, P24	−	+	−	+	+	+	−
P6, P74	−	+	−	+	− ^b^	+	−
P2, P4	−	+	−	+	+	−	−
P56, P75	−	+	−	+	−	−	−

^a^ truncated by IS*Pa26*; ^b^ an amplicon of only 340 bp was detected.

## Data Availability

All datasets are available. The four new class 1 integron sequences were submitted in GenBank (accession number): In2115 (OM831263), In2122 (OM831264), In2142 (OM863779), and In4879 (OQ858935).

## Supplementary Materials

The following supporting information can be downloaded at: https://www.mdpi.com/article/10.3390/antibiotics12091394/s1, Table S1: Characteristics of the 57 studied carbapenem resistant-*Pseudomonas aeruginosa* recovered from 43 patients from the Military Hospital of Tunis.
